# Engineered antibody cytokine chimera synergizes with DNA-launched nanoparticle vaccines to potentiate melanoma suppression *in vivo*

**DOI:** 10.3389/fimmu.2023.1072810

**Published:** 2023-02-23

**Authors:** Nicholas J. Tursi, Ziyang Xu, Michaela Helble, Susanne Walker, Kevin Liaw, Neethu Chokkalingam, Toshitha Kannan, Yuanhan Wu, Edgar Tello-Ruiz, Daniel H. Park, Xizhou Zhu, Megan C. Wise, Trevor R. F. Smith, Sonali Majumdar, Andrew Kossenkov, Daniel W. Kulp, David B. Weiner

**Affiliations:** ^1^ Vaccine and Immunotherapy Center, The Wistar Institute, Philadelphia, PA, United States; ^2^ Perelman School of Medicine, University of Pennsylvania, Philadelphia, PA, United States; ^3^ Inovio Pharmaceuticals, Bluebell, PA, United States

**Keywords:** cancer immunotherapy, antibody cytokine chimera, DNA vaccines, nanoparticle vaccines, melanoma

## Abstract

Cancer immunotherapy has demonstrated great promise with several checkpoint inhibitors being approved as the first-line therapy for some types of cancer, and new engineered cytokines such as Neo2/15 now being evaluated in many studies. In this work, we designed antibody-cytokine chimera (ACC) scaffolding cytokine mimetics on a full-length tumor-specific antibody. We characterized the pharmacokinetic (PK) and pharmacodynamic (PD) properties of first-generation ACC TA99-Neo2/15, which synergized with DLnano-vaccines to suppress *in vivo* melanoma proliferation and induced significant systemic cytokine activation. A novel second-generation ACC TA99-HL2-KOA1, with retained IL-2Rβ/γ binding and attenuated but preserved IL-2Rα binding, induced lower systemic cytokine activation with non-inferior protection in murine tumor studies. Transcriptomic analyses demonstrated an upregulation of Type I interferon responsive genes, particularly ISG15, in dendritic cells, macrophages and monocytes following TA99-HL2-KOA1 treatment. Characterization of additional ACCs in combination with cancer vaccines will likely be an important area of research for treating melanoma and other types of cancer.

## Introduction

Cancer is one of the leading causes of death worldwide. In 2018, there were an estimated 17 million new cancer cases and 9.5 million cancer deaths globally; these numbers are projected to almost double by 2040 (1). In the last decade, immunotherapy has demonstrated promise in the management of oncology patients, with checkpoint inhibitors and CAR T-cell therapy being examples of extremely successful approaches (2, 3). Immune checkpoint inhibitors target the PD-1 and CTLA-4 pathways to overcome a suppressive tumor microenvironment and potentiate endogenous anti-tumor T-cell immunity (2). Several agents including Ipilimumab (anti-CTLA-4) and Nivolumab (anti-PD-1) are effective in the management of patients with melanoma, small cell and non-small cell lung cancer (NSCLC), and renal cell carcinoma (RCC), and are FDA-approved first-line treatments for patients with advanced disease (4, 5).

Other immune interventions, such as the administration of cytokines and cancer vaccines, have also been explored in both pre-clinical and clinical studies and have shown efficacy to varying degrees (6, 7). IL-2 is an example of a cytokine which has been explored extensively over the past four decades and has been FDA-approved for the management of metastatic melanoma and RCC (8). In an early Phase II study, treatment of RCC patients with high dose (HD) IL-2 monotherapy led to complete and overall responses in 7% and 15% patients respectively (9). However, HD IL-2 treatment has been associated with significant toxicity, such as the potentially lethal Vascular Leak Syndrome (VLS), and did not significantly improve overall survival in cancer patients (10). Additionally, low dose (LD) IL-2 treatment preferentially saturates IL-2Rα (CD25), which is highly expressed on regulatory T cells (Tregs), over IL-2Rβ/γ, which is expressed on CD8+ T cells and NK cells; this paradoxically decreases anti-tumor immunity due to Treg activation (11). In response to such limitations, several studies have examined the introduction of specific mutations to decrease IL-2Rα binding to attenuate Treg activation (12, 13). One such example is Neo2/15, a *de novo* designed neoleukin with a sequence distinct from IL-2 and which possessed ablated binding to IL-2Rα yet retained binding to IL-2Rβ/γ (14). In a therapeutic mouse B16F10 melanoma model, Neo2/15 has been shown to synergize with tumor-specific antibody TA99 to improve survival. However, like wildtype IL-2, Neo2/15 was determined to have extremely short *in vivo* half-life (in the range of hours), warranting daily treatment of mice (14). Strategies that can augment engineered cytokine half-life may therefore enhance construct potency, and reduce the costs and complexity associated with daily dosing.

It has previously been described that multi-modality treatment may be important for eradication of large established tumors (15, 16). Particularly, a cancer vaccine capable of eliciting CD8+ T-cell immunity to tumor-associated antigens is a critical component of the regimen. While developments of vaccines capable of eliciting CD8+ T-cell (CTL) responses in people are historically challenging, few approaches, including viral-vectored vaccines (17), Dendritic Cell vaccines (18) and DNA vaccines (19), have been demonstrated to induce consistent CTL responses in the clinic. We have previously demonstrated that adaptive electroporation mediated DNA delivery can enable direct *in vivo* production of nanoparticle vaccines (DLnano vaccines) to elicit strong anti-tumor CTL responses by engaging antigen presenting cells (APCs) to mediate cross-presentation (20, 21).

In the present work, we evaluated the combined use of DLnano-vaccines with engineered novel immunotherapies in the management of melanoma. To improve half-lives of engineered cytokine constructs and simplify the dosing regimen, we designed antibody-cytokine chimeras (ACC) to scaffold engineered cytokines on the C terminus of antibody constant heavy chains, enabling simultaneous expression and purification of both tumor-specific antibody and cytokine from a single transfection. We confirmed expression of engineered ACC TA99-Neo2/15 and its binding to both tumor-associated antigen Tyrp1 and IL-2Rβ/γ. A weekly combination therapy of anti-PD1, TA99-Neo2/15 and DLnano-vaccines against Gp100, Tyrp1, and Trp2 significantly improved survival in a therapeutic murine B16F10 melanoma model, inducing complete response in a subset of mice. However, *in vivo* administration of TA99-Neo2/15 was observed to be associated with potent transient induction of pro-inflammatory cytokines, including IL-6 and TNFα. We hypothesized unopposed IL-2Rβ/γ signaling may be associated with detrimental cytokine storm and engineered a second-generation IL-2 variant, HL2-KOA1 with attenuated and yet partially retained binding to IL-2Rα. Recombinant TA99-HL2-KOA1 possessed a comparable *in vivo* pharmacokinetic (PK) profile to TA99-Neo2/15 and induced significantly lower systemic activation of proinflammatory cytokines, and yet retained a similar therapeutic efficacy in the B16F10 melanoma model. We performed single cell RNA sequencing (scRNAseq) on tumor-infiltrating lymphocytes, which demonstrated activation of Type I interferon pathway, particularly upregulation of ISG15 in DCs, macrophages and monocytes of tumor-bearing mice receiving TA99-HL2-KOA1 treatment. The work highlights potential utility of ACC in synergy with CD8+ T-cell vaccines such as a DLnano-vaccine in treating melanoma and potentially other types of cancer. Exploration of additional designed ACC constructs and their respective immunological mechanisms of action will likely be important to generate improved clinical immunotherapeutic outcomes.

## Results

### TA99-Neo2/15 synergized with DLnano-vaccines (TriVax) to suppress *in vivo* melanoma proliferation

**Figure 1:**
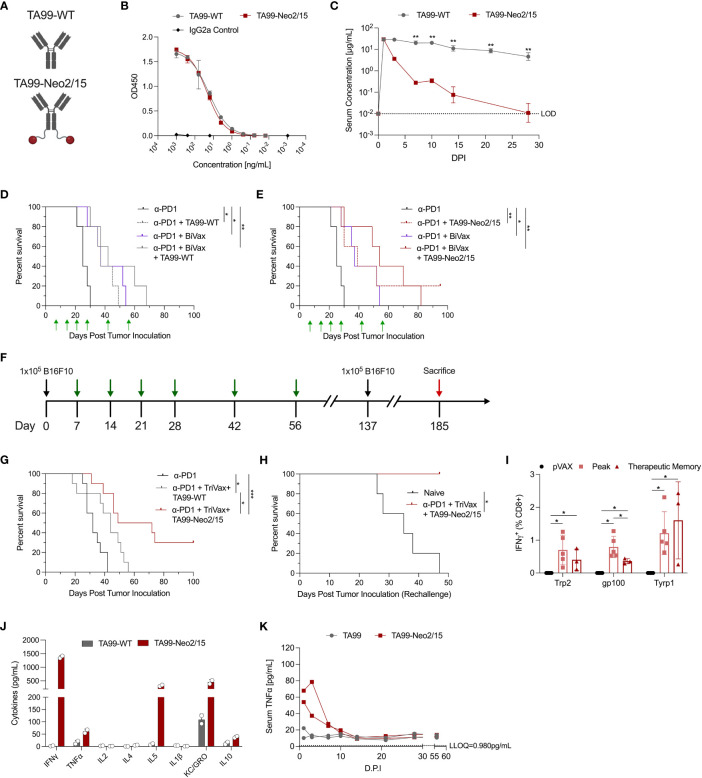
Pharmacokinetic and pharmacodynamic characterization of ACC TA99-Neo2/15. **(A)** Graphical representation of the TA99-WT and ACC TA99-Neo2/15; antibody is shown in grey and neoleukin Neo2/15 shown in red. **(B)** Binding of recombinant TA99-WT and TA99-Neo2/15 to recombinant human TYRP1 compared to a murine isotype IgG2a control. **(C)** Pharmacokinetic profiles of recombinant TA99-WT and TA99-Neo2/15 antibodies; serum at the indicated timepoints was assessed for TYRP1 binding by ELISA. **(D)** Survival curves of mice (n=5 per group) post tumor challenge following treatment with anti-PD1 alone (200µg), or anti-PD1 + TA99-WT (50µg), or anti-PD1 + BiVax (10µg each Trp2 and Gp100 DLnano vaccines), or anti-PD1 + TA99-WT + BiVax. 1x10^5^ B16F10 cells were subcutaneously administered into the right flank of C57BL/6 mice. Green arrows indicate administration of treatment. **(E)** Survival curves of mice (n=5 per group) post tumor challenge following treatment with anti-PD1 alone, or anti-PD1 + TA99-Neo2/15 (50µg), or anti-PD1 + BiVax, or anti-PD1 + TA99-Neo2/15 + BiVax. Challenge was performed as in **(D)**. **(F)** Timeline of tumor challenge. Black arrows indicate inoculation of 1x10^5^ B16F10 cells subcutaneously into the right flank of C57BL/6 mice. Green arrows indicate administration of treatment. Surviving mice were re-challenged subcutaneously in the opposite flank with 1x10^5^ B16F10 cells 137 days post initial tumor inoculation. Mice that survived rechallenge were sacrificed (red arrow) 48 days post rechallenge for analysis of cellular responses. **(G)** Survival curves of mice (n=10 per group) post tumor challenge following treatment with anti-PD1 alone (200µg), or anti-PD1 + TA99-WT (50µg) + TriVax (10µg each Trp2, Gp100 and Tyrp1 DLnano vaccines), or anti-PD1 + TA99-Neo2/15 (50µg) + TriVax. 16.7% of mice distributed across all groups had palpable tumors on Day 9. **(H)** Survival curves following rechallenge of mice that survived the initial tumor challenge in **(G)** (n=3 mice) or naïve mice that also received 1x10^5^ B16F10 cells at the rechallenge timepoint. **(I)** IFNγ+ CD8+ T cell responses against Trp2, Gp100, and Tyrp1 at both the peak and memory timepoints or in pVAX control vaccinated mice as determined by ICS. For determination of “Peak” response, C57BL/6 mice were immunized twice two weeks apart before being sacrificed 7 days post second immunization for assessment of cellular responses. Mice in the therapeutic “Memory” rechallenge in **(H)** (n=3 mice) were sacrificed 48 days post rechallenge for assessment of cellular responses. **(J)** Assessment of proinflammatory cytokines in two pools of mice sera Day 1 post administration of 100µg recombinant TA99-WT or TA99-Neo2/15. **(K)** Timecourse of serum TNFα levels in sera of mice administered antibody constructs as in **(J)**. Two pools of sera were used for panels **(J, K)**. Error bars represent standard deviation; non-parametric Mann-Whitney T test used to compare groups in **(C, I)**; log-rank test was used to compare between differences in all survival curves. The anti-PD1 and anti-PD1 + BiVax groups are duplicated on panels **(D, E)** for clarity. *p < 0.05, **p < 0.01, ***p < 0.001.

We designed ACC by engineering genetic fusion of a cytokine (such as the IL-2 mimetic Neo2/15) on the C-terminus of the constant heavy chain of a tumor-specific anti-Tyrp1 antibody TA99 (**Figure 1A**) (22). On a reducing SDS-PAGE gel, *in vitro* expressed TA99-Neo2/15 and wildtype TA99 (TA99-WT) featured identical migration of light chains but TA99-Neo2/15 heavy chains migrated at a higher molecular weight due to the incorporation of the Neo2/15 domain (**Supplementary Figure 1A**). Recombinant TA99-Neo2/15 purified using protein G was evaluated using size exclusion chromatography and showed a single species demonstrating homogenous dimeric pairing of designed antibody chains (**Supplementary Figure 1B**). Additionally, we determined that TA99-Neo2/15 binds to the melanoma associated antigen Tyrp1 almost as effectively as TA99-WT on a binding ELISA (**Figure 1B**). Next, we evaluated the *in vivo* pharmacokinetic properties of TA99-Neo2/15 in terms of its clearance and distribution. Using a Tyrp1-based quantification ELISA, we determined TA99-WT and TA99-Neo2/15 have similar peak serum concentration and TA99-Neo2/15 was cleared more quickly *in vivo* than TA99-WT with a half-life (t_1/2_) of 20 hours; we hypothesize this is potentially due to decreased Fc-FcRn interaction (**Figure 1C**). This is significantly longer than that of unconjugated Neo2/15, for which activity was no longer detected 8 hours post administration (14). To understand the *in vivo* distribution of TA99-Neo2/15, we fluorescently conjugated the engineered ACC to VivoTag680XL and passively administered 2nmol (of fluorophore) to B16F10-Luc tumor-bearing mice 9 days post tumor inoculation and imaged the mice with *In Vivo* Imaging System (IVIS) one day post fluorophore administration (**Supplementary Figure 1C**). We observed similar distribution patterns of fluorescently labelled TA99-Neo2/15 with fluorescently labelled murine IgG2a isotype control antibodies diffusely in vascular rich areas beneath the skin (ears, feet, tails, and flanks). Fluorescent signals from these labelled antibodies also co-localize with luminescence signals from the B16F10-Luc tumor, suggesting potential distribution of the antibody into the tumor sites (**Supplementary Figure 1C**). However, the specificity of the antibody component of an antibody-cytokine conjugate may not contribute to overall efficacy as previously demonstrated (23).

We further evaluated effectiveness of TA99-Neo2/15 in suppressing melanoma growth using the murine B16F10 model. Mice received a sub-cutaneous injection of 10^5^ B16F10 cells in the flank and were treated with anti-PD1 alone, anti-PD1 plus TA99-WT or TA99-Neo2/15, or anti-PD1 in combination with TA99-WT or TA99-Neo2/15 and DLnano-vaccines against Trp2 and Gp100 on D7, 14, 21, 28, 42 and 56 days post tumor inoculation. Previously, using DLnano-vaccines and anti-PD1 treatment in the therapeutic model without ACC, all mice succumbed to B16F10 melanoma by 35 days post tumor inoculation (20). Without the use of DLnano-vaccines, we observed similar median survival in mice treated with TA99-WT or TA99-Neo2/15 (**Figures 1D, E**). However, 20% of mice (one of five) in the TA99-Neo2/15 group exhibited a complete response and was tumor-free through the end of the study. DLnano-vaccines against Trp2 and Gp100 (BiVax) were shown to synergize with the immunotherapies, particularly TA99-Neo2/15, in extending median survival (**Figures 1D, E**). In the presence of DLnano-vaccines, TA99-Neo2/15 was observed to improve median survival of mice relative to TA99-WT alone, even though the study did not have sufficient power to detect differences between the two arms with n=5 per group (**Figures 1D, E**).

We sought to optimize the regimen by incorporating an additional novel DLnano-vaccine against Tyrp1 (TriVax). Similar to the other two DLnano-vaccines which we have previously reported (20), DLnano_LS_Tyrp1_455-463_ was shown to assemble homogeneously into nanoparticles by size exclusion chromatography (**Supplementary Figure 2A**). We increased statistical power of the study by including 10 animals in each arm, and down-selected to compare the two best performing groups in the preliminary study (anti-PD1 + TriVax + TA99-WT versus anti-PD1 + TriVax + TA99-Neo2/15) (**Figures 1F–H**). In this challenge study, we observed that mice treated with TA99-Neo2/15 exhibited significantly slower tumor growth (**Supplementary Figure 2B**), and statistically significant improved median survival. Tumor growth was slightly delayed with 16.7% of mice across all groups having a palpable tumor on Day 9, but all control mice succumb by Day 42. Despite B16F10 being a challenging melanoma model with limited mice clearing tumors in multiple immunotherapy settings (24, 25), our combined immunotherapy regimen increased the frequency of complete responders up to 30% in B16F10 challenged-mice (**Figure 1G**). To test the nature of protection, complete responders were rechallenged with 10^5^ B16F10 cells subcutaneously 81 days post the final vaccination. These animals fully rejected the tumors post rechallenge with no tumor growth or morbidity, in comparison to naïve mice which completely succumbed (**Figure 1H** and **Supplementary Figure 2C**). In parallel, another group of mice received 2 rounds of TriVax on D0 and 14 and were euthanized on D21 for determination of the peak cellular responses. At the memory timepoint, as compared to peak responses in mice, the rechallenge mice had similar CD8+ T-cell responses against Trp2 and Tyrp1 epitopes and slightly decreased response to Gp100 epitope (**Figure 1I**), demonstrating effective long-lasting memory T-cell responses induced by this regimen. Together, these data highlight the ability of ACC to synergize with anti-tumor DLnano-vaccines to promote survival of mice in a B16F10 melanoma model.

### Passive administration of TA99-Neo2/15 led to transient systemic activation of Th1 and Th2 cytokines

The engineered ACC TA99-Neo2/15 appeared efficacious in suppressing *in vivo* melanoma proliferation with or without the combined use of TriVax (**Figures 1E, G**). We next sought to characterize the cytokine profile of ACC post administration. In humans, HD IL-2 administration can be associated with significant toxicity due to VLS and cytokine storm. Here, we evaluated the proinflammatory cytokine activation profiles in mice following passive administration of 100µg TA99-Neo2/15 or 100µg TA99-WT with an electrochemiluminescence-based technique, which allowed simultaneous quantification of 10 analytes (26). Due to the scarcity of mouse serum, sera within a group of mice receiving the same treatment were pooled at each timepoint for downstream analysis. One day post administration, we observed a pronounced increase in Th1 cytokines, such as IFNγ and TNFα, Th2 cytokines such as IL-5, and other cytokines including KC-GRO and IL10 in mice treated with TA99-Neo2/15 versus those treated with TA99-WT (**Figure 1J**). The peak level of serum IFNγ is comparable with mice treated with LPS to induce systemic inflammation (27, 28). Additionally, the level of TNFα, a key mediator of cytokine storm (29), remained persistently elevated for at least a week post administration (**Figure 1K**). Levels of IFNγ, IL-4, KC-GRO and IL-10 largely normalized within 7 days post injection (d.p.i), whereas the level of IL-5 did not normalize until 14 d.p.i (**Supplementary Figure 3**). Therefore, designs for additional ACC capable of suppressing *in vivo* tumor growth without causing significant systemic activation of proinflammatory cytokines were investigated.

### Engineered TA99-HL2-KOA1 synergized with TriVax to suppress *in vivo* melanoma proliferation without causing significant systemic cytokine activation

**Figure 2:**
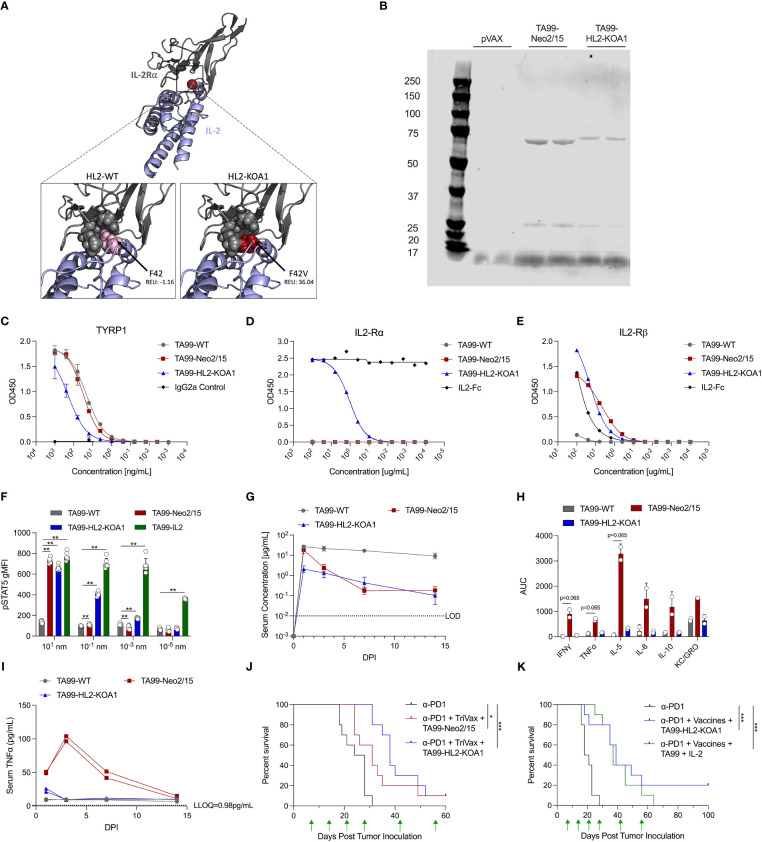
Design, pharmacokinetic and pharmacodynamic profiles of novel ACC TA99-HL2-KOA1. **(A)** Model depiction of engineered cytokine mimic HL2-KOA1 binding to IL-2Rα. IL-2 shown in blue, IL-2Rα shown in grey. Positions in contact with F42 shown as grey spheres. F42 and F42V shown as pink and red spheres respectively. Clash scores provided in total Rosetta energy units (REU). **(B)** Reducing SDS-PAGE analysis comparing the migration patterns of TA99-WT, TA99-Neo2/15, TA99-HL2-KOA1, pVAX backbone control transfection supernatants. **(C)** Binding of recombinant TA99-WT, TA99-Neo2/15, and TA99-HL2-KOA1 to recombinant human Tyrp1 compared to a murine isotype IgG2a control. **(D)** Binding of recombinant TA99-WT, TA99-Neo2/15, and TA99-HL2-KOA1 to recombinant human IL2-Rα compared to human IL2-Fc control. **(E)** Binding of recombinant TA99-WT, TA99-Neo2/15, and TA99-HL2-KOA1 to recombinant human IL2-Rβ compared to human IL2-Fc control. **(F)** Geometric mean fluorescence intensity of phosphorylated STAT5 in CD25+ CD4+ CD19- T cells from naïve mice (n=6) treated with indicated ACC. **(G)** Pharmacokinetic profile of recombinant TA99-WT and TA99-Neo-2/15 antibodies; C57BL/6 mice were injected intraperitoneally with 100µg of ACC constructs (n=5 mice per group). Serum at the indicated timepoints was assessed for TYRP1 binding by ELISA. **(H)** Total serum cytokine level over 14-day period in terms of area under the curve (AUC) in mice treated with ACC. **(I)** Timecourse of serum TNFα levels in sera of mice administered 100µg ACC. Two sera pools were used for **(H, I)**. **(J)** Survival curves of mice (n=10 mice per group) post tumor challenge following treatment with anti-PD1 alone, or anti-PD1 (200µg) + TA99-Neo2/15 (50µg) + TriVax (10µg each of Trp2, Gp100 and Tyrp1), or anti-PD1 (200µg) + TA99-HL2-KOA1 (50µg) + TriVax. 1x10^5^ B16F10 cells subcutaneously into the right flank of C57BL/6 mice on Day 0. Green arrows indicate administration of treatment. **(K)** Survival curves of mice (n=10 mice per group) post tumor challenge following treatment with anti-PD1 alone, or anti-PD1 (200µg) + TA99-HL2-KOA1 (50µg) + TriVax, or anti-PD1 + TA99 (50µg) + Human IL2 (5µg) + TriVax. Challenge and treatment scheme was performed as in **(J)** Error bars represent standard deviation; non-parametric Mann Whitney T test compared with TA99-WT used in **(F)**; non-parametric Kruskal-Wallis ANOVA was used in **(I)**; log-rank test was used to compare between differences in all survival curves; *p<0.05, **p<0.01, ***p<0.001.

We hypothesized the unopposed IL-2Rβ/γ activity of Neo2/15 in the complete absence of IL-2Rα-mediated Treg activity might contribute to the observed systemic activation of proinflammatory cytokines (30, 31). Indeed, in some studies CD25+ Treg depletion was known to exacerbate the toxicity of IL-2 treatment, including VLS, in mice (30, 32). We therefore engineered a second-generation IL-2 mimetic, HL2-KOA1, through structure-based design, by introducing a single point mutation F42V to decrease but not completely ablate IL2 IL-2Rα interaction, and to simultaneously preserve the IL2 IL-2Rβ/γ binding interface (**Figure 2A**). The higher Rosetta energy units (REU) of mutant HL2-KOA1 at position 42 demonstrate that the F42V mutation creates clashes with IL-2Ra as well as reducing many of the contacts that F42 maintained with positions M25, N27, L42 and Y43 of IL-2Rα. ACC TA99-HL2-KOA1 was designed in the same fashion as TA99-Neo2/15 with the genetic fusion of a cytokine mimetic on the C-terminus of the TA99 constant heavy chain. TA99-HL2-KOA1 was expressed *in vitro* and analyzed with reducing SDS-PAGE (**Figure 2B**), where we observed comparable migration patterns for both the heavy and light chains of TA99-HL2-KOA1 versus TA99-Neo2/15. Binding ELISA showed TA99-HL2-KOA1 was capable of binding to Tyrp1, albeit with a slightly lower affinity than that of TA99-WT or TA99-Neo2/15 (**Figure 2C**). Next, we assessed binding of TA99-WT, TA99-Neo2/15, TA99-HL2-KOA1, and human IL2-Fc (IL2-Fc) to human IL-2Rα and IL-2Rβ by binding ELISA (**Figures 2D, E** and **Supplementary Figure 4A**). While TA99-Neo2/15, TA99-HL2-KOA1, and IL2-Fc all bound to IL-2Rβ, TA99-Neo2/15 demonstrated complete lack of binding to IL-2Rα, as previously reported (14). TA99-HL2-KOA1 binds to IL-2Rα with significantly lower affinity than IL2-Fc, which required serial dilution by 10^12^-fold to get a full binding curve (**Supplementary Figure 4A**), over 4 orders of magnitude (EC_50_ of 1.098µg/mL for TA99-HL2-KOA1 versus EC_50_ of 0.00089µg/mL for IL2-Fc). To assess whether TA99-HL2-KOA1 demonstrates an intermediate activation phenotype consistent with IL-2Rα (CD25) binding data, we assessed STAT5 phosphorylation of splenocytes from naïve C57BL/6 mice by flow cytometry 30 minutes after incubation with TA99-WT, TA99-Neo2/15, TA99-HL2-KOA1, or TA99-IL2, which displays unmutated IL-2. In CD25+ CD4+ T cells, pSTAT5 gMFI for the TA99-HL2-KOA1 bound cells exhibits an intermediate phenotype, between TA99-Neo2/15 and TA99-IL2 (**Figure 2F**). Taken together, these data demonstrate TA99-HL2-KOA1 is an engineered intermediate between TA99-Neo2/15 and wild-type IL-2.

We next assessed the PK profile of TA99-HL2-KOA1 in mice receiving passive transfer of 100µg of recombinant protein. While the peak level of TA99-HL2-KOA1 in the serum was lower than those of TA99-WT or TA99-Neo2/15 1 d.p.i, clearance of TA99-HL2-KOA1 appeared to be slower than that of TA99-Neo2/15 such that serum concentrations of TA99-HL2-KOA1 appeared similar to that of TA99-Neo2/15 3 d.p.i (**Figure 2G**). We also examined systemic proinflammatory responses in mice receiving passive transfer of 100µg TA99-WT, TA99-Neo2/15 and TA99-HL2-KOA1. Decreased levels of TNFα, IFNγ, IL4, IL5, IL6, IL10, and KC/GRO was observed in mice treated with TA99-HL2-KOA1 than those treated with TA99-Neo2/15 (**Figures 2H**, **I** and **Supplementary Figures 4B–I**). Transient small increases IFNγ, TNFα and IL-5 were fully resolved by 3 d.p.i in the case of TA99-HL2-KOA1, whereas the increase in IFNγ, TNFα, IL-5, IL-6 and IL-10 did not fully resolve until 7 to 14 d.p.i in the case of TA99-Neo2/15 (**Figure 2I** and **Supplementary Figures 4B-I**). Systemic upregulation of certain proinflammatory cytokines, such as IFNγ and TNFα, have demonstrated pathology, including tissue damage and cell death (33). As a reference for pathologic upregulation of cytokines in serum, a predictive marker for respiratory failure in COVID-19 patients include serum levels of IL-6 greater than >80 pg mL^-1^ and C-reactive protein (CRP) >97 mg L^-1^ (34–36); peak levels of TA99-Neo2/15 serum IL-6 are in excess of 100 pg mL^-1^. Additionally, for serum TNFα levels, approximately 32 pg mL^-1^ was observed in patients hospitalized with COVID-19 as well as to CAR-T patients with cytokine release syndrome (37). Interestingly, serum IL-2 levels were suppressed in mice treated with either TA99-HL2-KOA1 or with TA99-Neo2/15 as compared to mice treated with TA99-WT (**Supplementary Figures 3B, 4B**). These data highlight the reduced proinflammatory serum cytokine profile of TA99-HL2-KOA1 compared to TA99-Neo2/15.

We next compared the efficacy of TA99-HL2-KOA1 in synergy with TriVax (against Trp2, Gp100 and Tyrp1) in suppressing *in vivo* melanoma proliferation. The same challenge and treatment regimen was employed as before, where 10^5^ B16F10 cells were inoculated subcutaneously and combination treatments were administered on D7, 14, 21, 28, 42 and 56 post tumor inoculation. TA99-HL2-KOA1 and TA99-Neo2/15 combination therapy with TriVax suppressed *in vivo* melanoma proliferation (**Supplementary Figure 4J**). Importantly, we observed that overall survival of the mice treated with TA99-HL2-KOA1 was non-inferior to those treated with TA99-Neo2/15 (in combination with anti-PD1 and TriVax), highlighting the designed second-generation ACC TA99-HL2-KOA1 had both an improved safety profile and performed at least as well as TA99-Neo2/15 (**Figure 2J**).

To compare the therapeutic potency of ACC TA99-HL2-KOA1 as compared to TA99-WT and human IL-2 administered separately, we performed an additional challenge study (**Figure 2K**). Mice were administered anti-PD1 alone, a triple therapy regimen including TA99-HL2-KOA1, or a quadruple therapy regimen including TA99-WT and human IL-2 at an equimolar ratio as compared with TA99-HL2-KOA1. We observed that mice treated with both combination therapies exhibited extended survival as compared to mice treated with anti-PD1 alone (**Figure 2K**). Importantly, while there is not a survival difference between the two groups treated with combination therapies, 2/10 mice treated with TA99-HL2-KOA1 but none in the quadruple therapy regimen had progression-free survival 100 days post initial tumor inoculation. Together, these data illustrate the non-inferior therapeutic efficacy of a three-regimen strategy employing ACC.

### Combination of DLnano-vaccine (Trp2Vax) and immunotherapy increased the frequencies and effector functions of tumor-infiltrating CD4+ and CD8+ T cells

**Figure 3:**
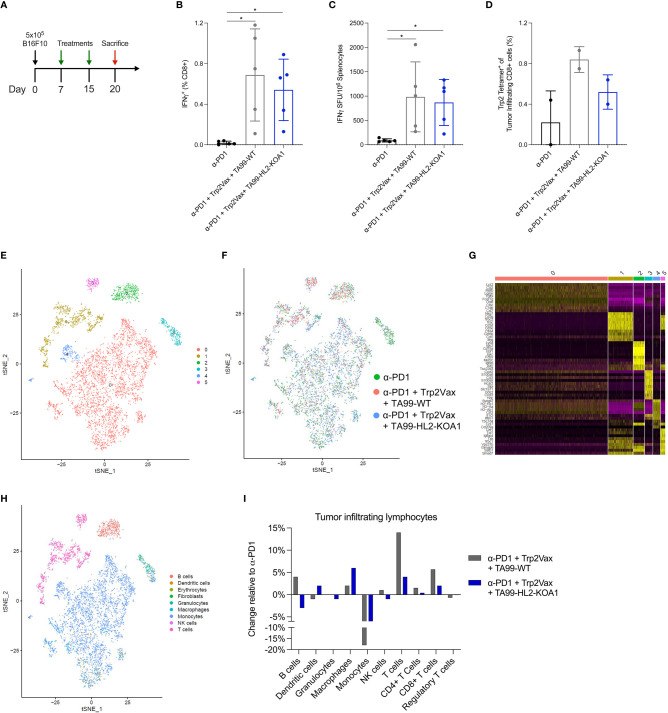
Single cell RNA sequencing revealed increased intratumoral infiltration of T cells following DLnano-vaccines and TA99-based immunotherapy treatments. **(A)** Tumor inoculation, treatment, and euthanasia scheme of the study. Mice received 5 x 10^5^ B16F10 cells subcutaneously on D0, and 2 treatments of either anti-PD1 only (200µg), anti-PD1 (200µg) + Trp2Vax (10µg of DLnano_LS_Trp2_188_) + WT TA99 (50µg) or anti-PD1 (200µg) + Trp2Vax + TA99-HL2-KOA1 (50µg) on D8 and 15, and were euthanized on D20 for spleen and tumor collection. **(B, C)** Trp2Vax induced Trp2-specific splenic IFNγ+ CD8+ T-cell responses as determined by ICS **(B)** and ELISpot **(C)**. **(D)** Frequency of Trp2-tetramer specific CD8+ T cells intratumorally in mice receiving each specified treatment. **(E)** TSNE plot demonstrating major clusters (0-5) of TILs isolated from three groups of mice after sample integration. **(F)** TSNE plot demonstrating distribution of TILs from each specimen amongst the main identified clusters from **(E)**. **(G)** Identification of top 10 most highly expressed genes in each cluster from the TSNE plot in **(E)**. **(H)** Assignment of each cluster with its respective cell type according to its gene signatures using SingleR and referencing mouseRNAseq database. **(I)** Comparison of the frequencies of each cell type amongst TILs from the three specimens. N=5 mice/group for **(A–C)**; Two groups mice were pooled for TIL analysis in **(D)**; N=5 mice/group were pooled for transcriptomic analyses in **(E–I)**. each dot represents a sampling point (individual animal, or individual pool of animal receiving same treatment). Error bar represents standard deviation. Two-tailed Mann-Whitney Rank test used to compare groups; p-values were adjusted for multiple comparison for **(B–D)**. *p<0.05.

Next, we explored the immunological basis for the improved therapeutic outcomes of mice treated with ACC TA99-HL2-KOA1 in a combination regimen. We first investigated vaccine-induced CD8+ T-cell responses in B16F10 tumor-bearing mice treated with anti-PD1, DLnano_LS_Trp2_188_ (Trp2Vax) and TA99-WT versus TA99-HL2-KOA1. Mice received 5x10^5^ B16F10 cells subcutaneously, two treatment regimens on D8 and 15 post tumor-inoculation and were euthanized 5 days post the final treatment (**Figure 3A**). We examined systemic Trp2-specific CD8+ T-cell responses in the spleens of vaccinated animals using a combination of intracellular cytokine staining (ICS) (**Figure 3B**) and IFNγ ELISpot assays (**Figure 3C**). In both cases, while we observed a significant increase in Trp2-specific CD8+ responses in animals receiving Trp2Vax in comparison to those receiving only anti-PD1, we did not observe that HL2-KOA1 treatment further augmented vaccine-induced CTL responses. In terms of CD4+ T-cell responses to the nanoparticle domain (38), we also observed similar vaccine-induced CD4+ responses in mice treated with TA99-WT or TA99-HL2-KOA1 using either ICS (**Supplementary Figure 5A**) or IFNγ ELISpot assays (**Supplementary Figure 5B**). Next, we compared Trp2-specific tumor-infiltrating CD8+ T-cells using Trp2 tetramer staining. Similarly, while we observed increased frequencies of Trp2-specific tumor-infiltrating CD8+ T-cells in mice receiving Trp2Vax, TA99-HL2-KOA1 treatment did not further increase frequency of this population (**Figure 3D**). We, therefore, hypothesized that there must be other types of tumor-infiltrating immune cells that mediated the therapeutic effect of TA99-HL2-KOA1 and adopted a transcriptomic approach to analyze the contributions of each individual tumor-infiltrating lymphocyte (TIL) sub-population more rigorously.

Due to cost considerations, we pooled TILs from all mice in each group receiving the same treatment and used MACS sorting to isolate viable cells prior to scRNAseq. Data were filtered and normalized to ensure robustness of transcriptomic analyses (**Supplementary Figures 5C, D**). We generated tSNE plot (**Figure 3E**) and visualized the distribution of cells for individual samples (**Figure 3F**) to ensure that the batch effects, if any, were corrected. To unambiguously assign each cluster with its respective cell type, we first identified the most highly expressed genes in each cluster (**Figure 3G**). Next, we performed unbiased cell-type recognition (**Figure 3H**) and further resolved the cluster identified as T cells into CD4+ and CD8+ T cells using CD4, CD8a and CD8b1 as markers (**Supplementary Figures 6A–C**). Then, we analyzed the frequencies of Tregs, as defined by CD4+ FoxP3+ cells (**Supplementary Figure 6D**). As compared to mice receiving only anti-PD1 treatment, we observed that Trp2Vax and TA99-WT or TA99-HL2-KOA1 treatment appeared to increase frequencies of tumor-infiltrating macrophages, CD4+ and CD8+ T cells, while decreasing the frequencies of monocytes (**Figure 3I**). As compared to TA99-WT treatment, treatment with TA99-HL2-KOA1 increased the frequency of macrophages by approximately 2-fold. Additionally, TA99-HL2-KOA1 treatment increased the frequency of dendritic cells by two-fold relative to anti-PD1 treatment alone, and four-fold relative to TA99-WT combination therapy. While HL2-KOA1 has residual IL-2Rα binding, frequency of tumor-infiltrating Tregs was not observed to be significantly elevated in mice treated with TA99-HL2-KOA1. In addition, we analyzed phenotypes of tumor-infiltrating CD8+ T cells by TOX, XCL1, IL7R, CD28, PRF1, and GZMB to further sub-categorize them as naïve, stem-like and terminally differentiated CD8+ T cells, as previously described (**Supplementary Figures 6E, F**) (39). TA99-HL2-KOA1 immunotherapy and Trp2Vax appeared to increase the frequencies of naïve tumor-infiltrating CD8+ T-cells but did not significantly change the frequencies of stem-like or terminally differentiated CD8+ T cells (**Supplementary Figure 6G**).

Next, we examined the transcriptomic features of key tumor-infiltrating effector cells (**Supplementary Figures 6H–J**) and antigen presenting cells (**Supplementary Figures 7A–D**) following immunotherapy and Trp2Vax treatments. To minimize non-specific findings, we examined genes that are differentially expressed with a significance of p<0.05 and those that are differentially expressed in both the TA99-WT and TA99-HL2-KOA1 combination therapy groups (relative to the anti-PD1 only group as the baseline). Using Reactome for pathway enrichment analyses (40), we observed Trp2Vax and immunotherapy treatment upregulated the IL-12 responsive genes, including ITGB1, SOCS3, IFNG and STAT4 in the tumor-infiltrating CD4+ T cells (with a False Discovery Rate, FDR= 0.0016) (**Supplementary Figure 6H**) (41). In CD8+ T cells, we observed upregulation of GZMA, which is indicative of effector functions, and CCR7, which is associated with increased homing to the tumor sites (**Supplementary Figure 6I**) (42). In NK cells, we observed upregulation of PRF1 (perforin), indicative of effector functions, and XCL1, indicative of active secretion of chemokines (**Supplementary Figure 6J**) (43). In tumor-infiltrating macrophages we also observed increased activation of cytokine-responsive genes IFITM3, CCR1 and UBB (FDR=10^-4^), as well as upregulation of genes involved in MHC Class II presentation H2-Aa, H2-Ab1, H2-Eb1 and CD74 (FDR=0.0015) (**Supplementary Figure 7C**) (44). Taken together, the data suggest combined immunotherapy and Trp2Vax treatment increased frequency and effector functions of tumor-infiltrating effector cells (CD4+ and CD8+ T cells an NK cells), and potentially activate APCs such as macrophages for antigen presentation.

Interestingly, we simultaneously observed downregulation of several HSF-1 responsive genes (**Supplementary Figure 8A**), including HSPA1A, HSPA1B, DNAJB1, HSPH1, HSPA8 and HSP90AA1 in multiple cell types, particularly B cells, CD8+ T cells, monocytes and NK cells, of mice receiving either TA99-WT combination therapy (**Supplementary Figure 8B**) or TA99-HL2-KOA1 combination therapy (**Supplementary Figure 8C**) relative to respective cell types in mice receiving only anti-PD1 treatment (45). The finding might be suggestive of decreased stress response in infiltrating immune cells due to prior immune modulation of the tumor microenvironment. Taken together, these data highlight the distinct immunomodulatory capability of DLnano vaccines together with antibodies or ACC on tumor-infiltrating lymphocytes.

### TA99-HL2-KOA1 treatment activated Type I interferon responses in tumor-infiltrating DCs, monocytes and macrophages

Finally, to elucidate the mechanism of action of TA99-HL2-KOA1, we examined differential gene expression of each major TIL population. For each cell type, we compared the gene expression profiles of TA99-HL2-KOA1 combination therapy treated sample versus anti-PD1 only or TA99-WT combination therapy treated samples. To avoid non-specific findings, we examined genes that are differentially expressed in both pairwise comparisons as aforementioned, and those with statistical significance (p<0.05). For CD8+ T cells and NK cells, we did not observe genes that were consistently up- or down-regulated in both pairwise comparisons, and those that can be mapped to a specific biological pathway of significance. For Tregs, we observed downregulation of genes involved in mitotic spindle formation and cell cycle progression, including TUBB4B, RCC2, and EML4 (FDR=0.005) (**Supplementary Figures 8A, B**) (46), indicating that TA99-HL2-KOA1 treatment did not induce active Treg proliferation, consistent with our previous observations (**Figure 3I**).

**Figure 4:**
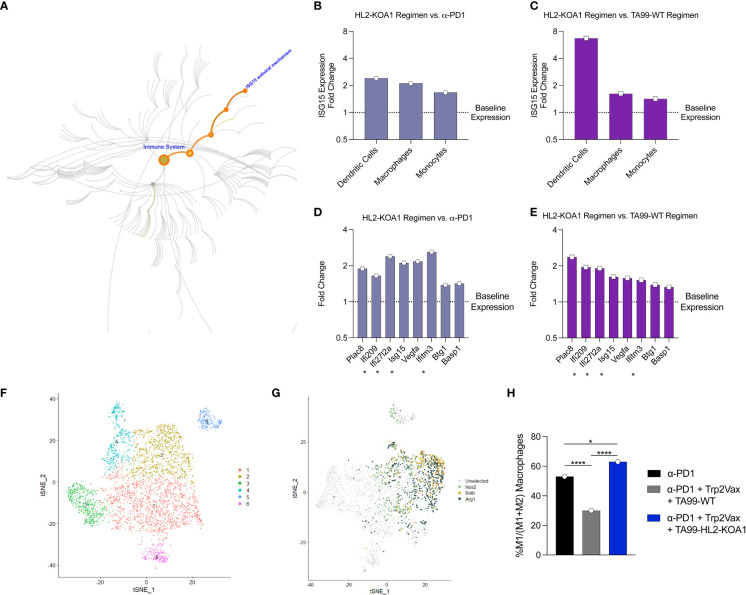
Transcriptomic analyses demonstrated upregulation of Type I interferon responses following TA99-HL2-KOA1 treatment. **(A)** Map of ISG15 as part of the Type I interferon responsive elements in pathway analysis. **(B, C)** Fold changes in ISG15 in intratumoral DCs, macrophages and monocytes in mice treated with anti-PD1+ Trp2Vax + TA99-HL2-KOA1 versus corresponding cell types from mice treated with only anti-PD1 **(B)** or with anti-PD1+ Trp2Vax + TA99-WT **(C)**. **(D, E)** Fold changes of the most upregulated genes in the intratumoral macrophages of mice treated with anti-PD1+ Trp2Vax + TA99-HL2-KOA1 versus intratumoral macrophages from mice treated with only anti-PD1 **(D)** or with anti-PD1+ Trp2Vax + TA99-WT **(E)**. Asterisk * marks IFN-responsive genes. **(F)** Re-clustering of cells as in **Figure 3E** based on canonical macrophage markers. **(G)** Nos2 and Arg1 expression clustering based on **(F)**. **(H)** Percent M1 macrophages in each treatment group relative to the total of M1 and M2 macrophages. N=5 mice/group were pooled for transcriptomic analyses in this Figure. *p=0.021, ****p=10^-7^ (PD-1 vs. TA99 Regimen), ****p<10^-10^ (TA99 Regimen vs. HL2-KOA1 Regimen) by Fisher Exact Test. Each dot represents a pool of 5 animals receiving the same treatment.

We observed that the Type I interferon-responsive gene, ISG15, was consistently elevated following TA99-HL2-KOA1 treatment across multiple APC types examined (47), including DCs, macrophages and monocytes in both pairwise comparisons (**Figures 4A–C**). ISG15 has been demonstrated to inhibit cancer progression and is often dysregulated within cancers (48). Macrophages, in particular, demonstrate activation of additional Type I interferon responsive genes, including IFI209, IFI2712A, ISG15 and IFITM3 (FDR=10^-5^) (**Figures 4D, E**) (49), highlighting TA99-HL2-KOA1 may directly or indirectly through other cytokine mediators in the milieu engage innate immunity for anti-tumor responses. Additionally, we sought to determine whether intratumoral macrophages were polarized toward M1 macrophages in response to upregulation of Type I interferon responsive genes. As such, cells that were predicted to be macrophages were re-clustered (**Figure 4F**). We then defined M1 and M2 macrophages based on the expression of Nos2 and Arg1 respectively. Macrophage populations can be readily defined within cluster 2 (**Figure 4G**). Among M1 and M2 macrophages, the total proportion of M1 macrophages decreased with TA99-WT combination therapy treated relative to anti-PD1 alone. However, compared to both anti-PD1 only or TA99-WT combination therapy, the TA99-HL2-KOA1 regimen increased the proportion of M1 macrophages (**Figure 4H**). Together, these data show the capacity of TA99-HL2-KOA1 to uniquely induce innate immune anti-tumor responses when used in a combination therapy approach.

## Discussion

Our work demonstrates the utility of ACC in combination with DLnano-vaccines in suppressing in vivo melanoma proliferation. While some preclinical and clinical studies have explored ACC in the treatment of cancer, such as the use of anti-GD2 IL2 conjugate Hu14.18-IL2 in the treatment of neuroblastoma and melanoma (50), this study demonstrates that next-generation engineered cytokine such as Neo2/15 and a novel designed IL-2 variant HL2-KOA1 can also be conveniently and robustly conjugated to a tumor-specific antibody downstream of the CH3 domain of the constant heavy chain. Such a design will enable convenient simultaneous expression and purification of both antibody and cytokine mimetic from a single transfection using a Protein G column, without the need of *post-hoc* bi-component assembly (51). Additionally, whole IgG-cytokine conjugate may demonstrate improved therapeutic half-life as compared to scFV-cytokine conjugate due to Fc-FcRn recycling (52). In addition, conjugation allows the possibility of three regimen therapy which performed as well as, if not better, than four regimen therapy (**Figure 2K**) with reduced cost. We did observe, in our case, relatively shorter half-life of TA99-Neo2/15 and TA99-HL2-KOA1 in comparison with TA99-WT as steric hindrance by the cytokine domain on the C-terminus of the antibody heavy chain may partially impede Fc-FcRn interaction. Further work to evaluate additional variants of ACC, including those with different linker lengths between the antibody and cytokine domains, may be important to determine if the therapeutic half-lives of ACC may be further extended to decrease dosing requirements. Importantly, this work demonstrates synergy between ACC and a CTL activating vaccine, such as a DLnano-vaccine, to improve therapeutic responses, and in some scenarios achieve complete remission and durable protection, in the management of melanoma. The durability of protection was extended to enable survival post rechallenge 81 days after initial tumor implantation. To determine the role of prior tumor exposure in the observed extended protection, future studies to examine responses to additional antigens not included in the vaccine regimen are warranted. The B16F10 model is an aggressive, well-validated model for murine melanoma studies (53, 54). While we have directly evaluated DLnano-vaccine in this context, it may be conceivable that other cancer vaccines, such as peptide vaccines (55), DC vaccines (18), viral-vectored vaccines (56), and amphiphilic vaccines (57) may also be combined with ACCs in the treatment of cancer.

While we have examined the pharmacodynamic property of ACC and its efficacy in suppressing *in vivo* melanoma proliferation, we have also characterized its pharmacokinetic properties, including its distribution, rate of clearance, and safety profile. The use of electrochemiluminescence assays may be a powerful tool to simultaneously interrogate multiple plasma cytokine analytes at various timepoints; a careful analysis of proinflammatory cytokines such as TNFα and IL-6 can help predict potential adverse effects to the treatment regimen. For the first-generation ACC TA99-Neo2/15, we observed a robust induction of proinflammatory Th1 cytokines (IFNγ, TNFα), Th2 cytokines (IL-4, IL-5, IL-6) and anti-inflammatory cytokine IL-10. While prior work with Neo2/15 demonstrated it had lower toxicity than IL-2 (14), the increased adverse effects observed in our study might have been due to the prolonged half-life of ACC TA99-Neo2/15 (20 hours versus less than 8 hours). We hypothesized the pronounced cytokine induction of TA99-Neo2/15 may be due to its unopposed activity on IL-2Rβ/γ with completely absent activity on IL-2Rα (CD25) (32). Indeed, following HD recombinant IL-2 treatment, BALB/c mice, which have increased CD25+ Treg activity, manifest fewer symptoms than C57BL/6 mice. Systemic depletion of CD25+ Tregs were demonstrated to exacerbate the adverse effects of HD IL-2 treatment, including VLS (30). Correspondingly, treatment with the second-generation ACC TA99-HL2-KOA1, which preserved IL-2Rβ/γ binding and IL-2Rα binding to a small extent, induced attenuated inflammatory cytokine responses without compromising therapeutic efficacy. Additional strategies to decrease systemic toxicity of IL-2 conjugated antibodies may also be considered. For example, incorporation of coil-coiled domain with a matrix metalloproteinase (MMP) cleavable site may allow IL-2 to selectively exert its action intratumorally, where there is a high local concentration of MMP (58).

We employed transcriptomic analyses on TILs to determine the immunological basis for the enhanced protection observed with TA99-HL2-KOA1; this was the first transcriptomic analysis of the immune response elicited from a DLnano vaccine. The ACC treatment was not shown to increase vaccine-induced tumor antigen-specific CTL responses. Despite residual IL-2Rα binding of HL2-KOA1, we did not observe increased frequency of Tregs, and in fact observed slight downregulation of some genes involved in mitosis and proliferation. We also did not observe increased frequencies of tumor infiltrating CD4+, CD8+ and NK cells following ACC treatment. Further investigation as to the mechanism for the decrease of NK and effector T cells, particularly CD8+ T cells, is warranted. Importantly, upregulation of Type I interferon responsive genes, especially ISG15, was observed in major tumor infiltrating APCs, particularly DCs and macrophages. ISG15 is a small secreted protein induced by Type I interferon signaling and can potentiate IFNγ secretion by lymphocytes and serve many anti-viral functions (47). In the cancer setting, extracellular ISG15 acts as an immune adjuvant to enhance antigen specific CD8+ T cell tumor immunity, increasing their production of IFNγ (59). Specifically, a subset of tumor-associated macrophages can secrete Type I interferons such as IFNγ, IFNβ, and IFNα within the local tumor environment (60, 61). It remains to be determined whether the TA99-HL2-KOA1 exerts direct effects on macrophages and DCs to potentiate Type I interferon responses, or the responses were initiated by other proinflammatory cytokines in the milieu. However, a global polarization of intratumoral macrophages toward M1 occurred with TA99-HL2-KOA1 treatment. M1 macrophages are known to mediate tumor suppression through antibody-dependent cellular cytotoxicity as well as direct cytotoxicity toward tumor cells by release of reactive oxygen species and nitric oxide among others (62, 63). Additionally, a high M1 to M2 ratio was associated with improved overall and progression-free survival in a prospective study of ovarian cancer patients (64). These observations were consistent with a preliminary human study, in which transcriptomic changes in PBMCs and TILs were assessed in melanoma patients following IL-2 treatment. The study reported IL-2 to have a minimal effect on activation, proliferation, and migration of T cells, but altered gene expression profiles of mononuclear cells and led to upregulation of interferon-responsive genes (65). Further understanding of the effector profile of both intratumoral M1 macrophages as well as antigen-specific CD8+ T cells is warranted. Additional transcriptomic analyses will also be needed in the future to determine whether the ACC can have a direct impact on tumor cells, as well as a time course of the changes in the expression profiles of TILs.

It may be important to design and characterize additional ACCs with variations of tumor specific antibodies, linker domains, and engineered cytokines (IL-2, IL-12, IL-21 and IFNα among others). In each case, PD evaluations (binding of the engineered cytokine to its target receptor, *in vivo* efficacy of ACC treatment, and transcriptomic changes following ACC treatment) as well as PK assessments (half-lives, distribution, and induced cytokine profiles) will both be important to determine the safety and utility of the designed ACC. Additionally, next generation DLnano vaccines, such as those scaffolding neoantigen epitopes, may serve to further extend efficacy beyond established antigens for melanoma. Along with a cancer vaccine, such as a DLnano-vaccine, ACCs will be an important component of a therapeutic treatment regimen to consider and develop in the critical era of cancer immunotherapy.

## Materials and methods

### Design of DNA-launched nanoparticle vaccines

DLnano_LS_Trp2_188_ and DLnano_LS _Gp100_25_ were developed in a previous work (20). Similarly, DLnano_LS_Tyrp1_455_ was designed by scaffolding the C57BL/6 class I epitope (CTAPDNLGYM) to the C-terminus of modified DLnano_LS_GT8.

### Design of HL2-KOA1

Fastdesign was used (input PDB 2ERJ) to model the F42V point mutation in designed construct HL2-KOA1. Native rotamers were preserved. Rosetta modeling was used to compute Rosetta energy units and to visualize contacts and clashes. Structures were visualized in PyMOL.

### Transfection

2 x 10^6^ Expi293F cells (Invitrogen) per 1mL Expi293F Expression Medium were plated on 12 well plates. The following day, a total of 2µg of each antibody-cytokine chimera (1µg each HC and LC) were plated on cells formulated in OPTI-MEM (Invitrogen) and ExpiFectamine (Invitrogen) transfection reagent. Approximately 18 hours post transfection, transfection enhancers were added according to manufacturer’s specifications. Cells were maintained in 8% CO_2_ conditions at 37°C until harvesting at 3 days post transfection. Cells were then spun at 5000rpm for 10 minutes and supernatants were subsequently collected for future assays.

### Size exclusion chromatography

2 x 10^9^ Expi293F cells (Invitrogen) in 1L Expi Expression medium (Invitrogen) were transfected with 150 µg pVAX-1 plasmid vector encoding the TA99-Neo2/15 or TA99-HL2-KOA1 heavy chain and 150 µg pVAX-1 plasmid vector encoding TA99 light chain with PEI (Sigma Aldrich)/OPTI-MEM (Invitrogen) and harvested 6 days post-transfection. Transfection supernatant was first purified with affinity chromatography using the AKTA pure 25 system and Protein G column (GE Healthcare). The eluate fractions from the affinity purification were pooled, concentrated with Amicon Ultra-15 Centrifugal Filter Unit with 30kDa cut-off (Milipore), and dialyzed into 1X PBS buffer before being loaded onto the Superose 6 Increase 10/300 GL size-exclusion chromatography (SEC) column (GE healthcare) for purification. Identified eluate fractions were then collected and concentrated to 1 mg/mL in PBS as previously described (21).

### Immunoblotting

To detect the presence of antibody-cytokine chimera in transfection supernatant, approximately 10µL of sample was run on NuPAGE™ 4-12% Bis-Tris gels (Thermo Fisher Scientific) in MOPS buffer. Briefly, all samples were reduced by heating samples in the presence of sample LDS buffer and sample reducing agent (Thermo Fisher Scientific) for 10 minutes at 70°C before loading onto the gel. Subsequent to electrophoresis, samples were transferred to a PVDF membrane and blocked for 1 hour at room temperature (RT) with Odyssey Blocking Buffer (OBB; LI-COR). Membranes were stained using 1:1000 IRDye 800CY goat anti-mouse IgG (LI-COR) formulated in OBB with 0.1% Tween-20 and 0.1% SDS at RT for 1 hour. Subsequently, membranes were washed with 0.05% Tween-20 in PBS before a PBS rise. Membranes were scanned using the LI-COR Odyssey CLx.

### Study approval

All animal studies were conducted under protocol 201221 and 201410 approved by the Wistar Institute Institutional Animal Care and Use Committee (IACUC).

### Tumor challenge model

Six to eight week old female C57BL/6J mice (Jackson Laboratory) were housed in the Wistar Institute Animal Facility. B16F10 cells (ATCC, Catalog: CRL-6475; authenticated by ATCC) were maintained using D10 media consisting of 10% FBS (Lampire) in DMEM (Corning) under low passage (<10). Cells were routinely tested for mycoplasma as well as other mouse pathogens. On the day of tumor inoculation, cells were trypsinized and filtered through a 70µm strainer to generate a single-cell suspension. Tumor cells were formulated in PBS (1 x 10^5^ cells per 100µL PBS) and administered subcutaneously on the left flank to mice. Tumor size was recorded every two days with a digital caliper, and tumor volume was calculated using the formula V=0.5W^2^L (V= volume, W= width, L= length). A tumor volume ≥ 2000mm^3^ or length/width ≥ 20mm^3^ was considered a humane endpoint, and mice were euthanized according to the established Wistar IACUC protocol. For recombinant anti–PD-1 (RMP1-14, Bio X Cell, Catalog: BE0146) administration, 200 µg of antibody was injected intraperitoneally formulated in 100 µL PBS to each mouse weekly beginning 7 days post tumor inoculation. Treatments became bi-weekly and stopped on Day 56 for all studies. For studies using DLnano_LS_Trp2_188_ and DLnano_LS _Gp100_25_, mice were immunized with 10µg of each vaccine into both tibialis anterior muscles. For studies using DLnano_LS_Trp2_188_, DLnano_LS _Gp100_25_, and DLnano_LS_Tyrp1_455_, mice were immunized with 10µg of each vaccine construct into both tibialis anterior muscles as well as the right quadricep. All mice received intramuscular adaptive electroporation with the CELLECTRA 3P device (Inovio Pharmaceuticals). For mice that received recombinant antibody-cytokine chimera or TA99, 50µg of protein was administered intraperitoneally formulated in 100µL PBS. For mice treated with combination therapy TA99 plus human IL2, equimolar ratios of 50µg TA99 and 5µg recombinant human IL-2 (Gibco, Catolog # PHC0023) were administered intraperitoneally in 100µL PBS.

### Pharmacokinetics studies

For the pharmacokinetics experiments, six to eight week old female C57BL/6J mice (Jackson Laboratory) were administered 100µg of each recombinant antibody-cytokine chimera intraperitoneally suspended in 100µL PBS. Intermandibular bleeds were taken on Day 0, 1, 3, 7, and 14 post intraperitoneal injection. Blood was centrifuged and sera was stored at -20°C until time of assay.

### *In vivo* imaging study

B16F10-Luc2 cells (ATCC, Catalog: CRL-6475-LUC2; authenticated by ATCC) were passaged in maintained using D10 media consisting of 10% FBS (Lampire) in DMEM (Corning) enriched with 10 µg/mL blasticidin (Gibco). For the tumor challenge, mice were administered 150 mg/kg of VivoGlo™ Luciferin (Promega) formulated in sterile PBS, and subsequently imaged with an IVIS Spectrum CT for Bioluminescence with the auto-exposure settings (or 60 seconds, whichever was shorter) 10 minutes post injection. Antibody (TA99-Neo2/15 or mouse IgG2a isotype control) was labelled with VivoTag 680 XL Fluorochrome (Perkin Elmer, Catalog #: NEV11119) according to manufacturer’s instruction. Upon fluorescent conjugation, unreacted fluorochromes were removed using a ZEBA desalting column (ThermoFisher), and the degree of labelling was determined by measurements of the absorbance of the analyte at 280nm and 670nm wavelengths. 2.0nmol of fluorophore-conjugated antibody in 100µL PBS was administered to each tumor-bearing mouse *via* retro-orbital injection 8 days post tumor inoculation, and the mice were imaged with IVIS (auto-exposure setting for VivoTag 680XL fluorochrome) 24 hour after fluorophore-conjugated antibody treatment.

### ELISA

#### TYRP1 Binding ELISA

96-well half area plates (Corning) were coated with 1µg/mL recombinant human His-tagged TYRP1 (Sino Biologicals, Cat# 13224-H08H) overnight at 4°C. The following day, plates were blocked with 1x PBS containing 5% skim milk (Sigma), 10% goat serum (Milipore), 1% bovine serum albumin (BSA; Sigma), 1% fetal bovine serum (FBS, Lampire) and 0.2% Tween-20 (Sigma) for 2 hours at RT. Plates were subsequently incubated with serially diluted mouse sera or recombinant protein antibody-cytokine chimera (depending on assay) for 2 hours at 37°C before incubation with 1:20000 HRP-conjugated anti-mouse IgG H+L (Bethyl, Cat# A90-116P) for 1 hour at room temperature. In addition, mouse IgG2a (BioXcell, Catalog: C1.18.4) was used as an isotype control. Following this, plates were developed with TMB substrate (Thermo) for approximately 5 minutes at RT before being stopped with 2N H_2_SO_4_. Plates were read with the BioTEK Synergy 2 plate reader and absorbance measured at 450 and 570nm.

#### IL2-RA Binding ELISA

ELISA protocol the same as described in section TYRP1 Binding ELISA except that plates were coated with 2µg/mL recombinant mouse His-tagged IL2-RA (Sino Biologicals, Cat# 50292-M08H) overnight at 4°C. In addition, serially diluted recombinant protein variants of antibody-cytokine chimera or recombinant mouse Fc-tagged IL2 (IL2-Fc) (Molecular Innovations, Cat# MIL2-FC-0.05MG) were used in place of serially diluted mouse sera.

#### IL2-RB Binding ELISA

ELISA protocol the same as described in section TYRP1 Binding ELISA except that plates were coated with 2µg/mL recombinant mouse His-tagged IL2-RB (Sino Biologicals, Cat# 50792-M08H) overnight at 4°C. In addition, serially diluted recombinant protein variants of antibody-cytokine chimera or recombinant mouse Fc-tagged IL2 (IL2-Fc) were used in place of serially diluted mouse sera.

#### Proinflammatory cytokine ELISA

To quantify cytokine levels in sera, V-PLEX^®^ Plus Proinflammatory Panel 1 Mouse Kit (MSD; K15048G) was used according to manufacturer’s specifications. Briefly, lyophilized calibrator was reconstituted with manufacturer’s diluent and serially diluted. Serum samples were pooled and diluted 2 fold in diluent 41. Plates were washed with 0.05% Tween-20 in PBS (PBS-T) before incubation with calibrators and serum samples for 2 hours at RT shaking at 700rpm. Plates were washed again with 0.05% PBS-T and incubated with SULFO-TAG-conjugated anti-mouse antibodies to IFN-γ (Cat# D22QO-2), IL-1β (Cat# D22QP-2), IL-2 (Cat# D22QQ-2), IL-4 (Cat# D22QR-2), IL-5 (Cat# D22QS-2), IL-6 (Cat# D22QX-2), KC/GRO (Cat# D22QT-2), IL-10 (D22QU-2), IL-12p70 (Cat# D22QV-2), TNF-α (D22QW-2) diluted 1:60 in diluent 45 for 2 hours at RT shaking at 700rpm. Plates were subsequently washed and developed with 2x read substrates according to manufacturer’s instructions and and the electrochemiluminescence from each well was recorded on MESO SECTOR S 600.

#### ELISpot assay

Spleens were collected from C57BL/6 mice and homogenized into single-cell suspensions with a tissue stomacher in 10% FBS/1% penicillin-streptomycin (Sigma) in RPMI 1640. Red blood cells were subsequently lysed with ACK lysing buffer (ThermoFisher), and the percentage of viable cells were determined with Trypan Blue exclusion using Vi-CELL XR (Beckman Coulter). 200,000 cells were then plated in each well of mouse IFNγ ELISpot plates (MabTech), followed by addition of peptide pools that either span the lumazine synthase domain, or individual Trp2_188_ (SVYDFFVWL), Gp100_25_ (EGPRNQDWL) and Tyrp1_455_ (CTAPDNLGYM) peptides at 5 µg/mL of final concentration for each peptide (GenScript). The cells were then stimulated at 37°C for 16-18 hours, followed by development according to the manufacturer’s instructions. Spots for each well were then imaged and counted with ImmunoSpot Macro Analyzer.

### Intracellular cytokine staining

Single-cell suspension from spleens of immunized C57BL/6 mice were prepared as described before and stimulated with peptides that either span the lumazine synthase domain, or individual Trp2_188_ (SVYDFFVWL), Gp100_25_ (EGPRNQDWL) and Tyrp1_455_ (CTAPDNLGYM) peptides at 5 µg/mL for 5 hours at 37°C in the presence of 1:500 protein transport inhibitor (ThermoFisher) and anti-mouse CD107a-FITC (ThermoFisher). The cells were then incubated with live/dead Fixable Violet Dead Cell Stain Kit (for 405 nm excitation) for 10 minutes at room temperature, and surface stained (anti-mouse CD4-BV510, Biolegend, Catalog: 100559; anti-mouse CD8–APC-Cy7, Biolegend, Catalog: 100714) at room temperature for 30 minutes. The cells were then fixed and permeabilized according to manufacturer’s instructions for BD Cytoperm Cytofix kit and stained with anti-mouse IL2–PE-Cy7 (BioLegend, Catalog: 503832), anti-mouse IFNγ-APC (BioLegend, Catalog: 505810), anti-mouse CD3e–PE-Cy5 (BioLegend, Catalog: 100310), and anti-mouse TNFα-BV605 (BioLegend, Catalog: 506329) at 4°C for 1 hour. The cells were subsequently analyzed with LSR II 18-color flow cytometer. The data was analyzed with FlowJo V10.6.1.

### Phosflow

Single cell suspensions from spleens of un-immunized C57BL/6 mice were prepared as described previously and incubated with supernatant containing TA99, TA99-Neo-2/15, TA99-HL2-KOA1, or TA99-IL2 at indicated concentrations for 30 min at 37°C. Following stimulation, cells were washed and incubated with Live/Dead Fixable Aqua Dye, anti-mouse CD4-BV421, anti-mouse CD19-BV650, anti-mouse CD25-FITC (all from Biolegend) for 10 min at room temperature. The cells were then fixed using BD Cytofix (BD) and permeabilized using Perm Buffer III (BD) according to manufacturer’s protocol. Cells were then stained with anti-mouse pSTAT5-PE (ThermoFisher) for 30 min at room temperature. The cells were then resuspended in FACS buffer and acquired with a BD FACSymphony A3 flow cytometer.

### Tumor infiltrating lymphocyte isolation and Tetramer staining

Tumors were harvested and stored in 1mL RPMI 1640 (Corning) until time of processing. Tumors were dissociated using the Miltenyi Mouse Tumor Dissociation Kit according to manufacturer’s specifications. Briefly, dissociation enzymes were reconstituted using RPMI 1640 and formulated in RPMI 1640 in a gentleMACS™ C tube. Tumors were transferred into C tubes before dissociation using a gentleMACS™ Octo Dissociator. Samples were then incubated for 40 minutes at 37°C and vortexed at 5 minute intervals. Samples were again dissociated using the gentleMACS™ Octo Dissociator. Samples were resuspended and strained through a 70µm strainer, and strainers were washed with RPMI 1640. The percentage of viable cells was counted using Trypan Blue exclusion on a Vi-CELL XR (Beckman Coulter). Cells were resuspended in FACS buffer and incubated with mouse CD45 microbeads (Miltenyi, Cat# 130-052-301) at 4°C for 15 minutes. MS columns were then primed and rinsed with 1% FBS/PBS under a magnetic field before elution into clean collection tubes by removal of the beads from the magnetic field. TILs were subsequently plated into 96-well plates and stained with live/dead Fixable Violet Dead Cell Stain Kit (for 405 nm excitation) for 10 minutes at room temperature, washed and then simultaneously stained with PE-conjugated Trp2-H2-Kb tetramer (MLB, Catalog: TB-5004-1) at 1:20 dilution and APC anti-CD8 (GeneTax, Catalog: GTX76346) at 1:20 dilution in 1% FBS/PBS. The cells were subsequently washed and analyzed with LSR II 18-color flow cytometer.

### TIL single-cell 10x genomics cDNA library prep

TILs were first incubated with live/dead Fixable Violet Dead Cell Stain Kit (for 405 nm excitation) for 10 minutes at room temperature, washed, and then sorted on FACSAriaII for the isolation of viable cells. Viable single cells from each of the 3 mouse TIL samples were uniquely barcoded using the 10× chromium single-cell platform, and complementary DNA (cDNA) libraries were prepared for Next Generation Sequencing according to the manufacturer’s protocol (Chromium Next GEM Single Cell 3′ Reagent Kits v3.1, 10× Genomics, USA). Cell suspensions of each sample, reverse transcription master mix, and partitioning oil were loaded on a single-cell “G” chip with a targeted cell output of 6,000 cells per library and then run on the Chromium Controller. A total of 3 lanes were used on the G chip, 1 lane/sample. Reverse transcription was performed within the droplets at 53°C for 45 min and newly synthesized cDNA was amplified for 11 cycles on a Veriti Thermal Cycler (Thermofisher, USA). cDNA size selection was performed using SPRIselect beads (Beckman Coulter, USA) at a ratio of SPRIselect reagent volume to sample volume of 0.6. cDNA was analyzed on an Agilent Bioanalyzer High Sensitivity DNA chip (Agilent, USA) for qualitative and quantitative control purposes. cDNA was fragmented using the proprietary fragmentation enzyme blend for 5 min at 32°C, followed by end-repair and A-tailing at 65°C for 30 min. cDNA was double-sided size selected using SPRIselect beads. Sequencing adaptors were ligated to the cDNA at 20°C for 15 min. and after a round of post-ligation SPRIselect bead clean-up, cDNA was amplified for 15 cycles using a sample-specific index oligo as a primer. A final round of double-sided size selection using SPRIselect beads followed. Final library size and quantity was determined using an Agilent Bioanalyzer High Sensitivity DNA chip and a Qubit dsDNA High Sensitivity Assay kit (Thermofisher, USA), respectively. Additional library quantification was done using the Kapa Library Quantification kit for Illumina Libraries (Roche, USA). cDNA libraries were pooled in equimolar concentrations and sequenced on a NextSeq 500 Illumina platform using the 75bp High Output sequencing kit (Illumina, USA), aiming for 120 million reads per library and a sequencing configuration of 28 base pair (bp) on read1 and 55 bp on read2.

### ScRNA-seq cell data processing

The Cell Ranger Software Suite (Version 3.1.0, https://support.10xgenomics.com) was used to preprocess scRNA-seq data. Cellranger count was executed with refdata-cellranger-mm10-3.0.0 transcriptome to map reads on the mouse genome (mm10) using STAR [ref: https://pubmed.ncbi.nlm.nih.gov/23104886/] (Version 2.5.2b) and UMIs (unique molecular identifier) were counted. Seurat package (version 3.2.0) [ref: https://pubmed.ncbi.nlm.nih.gov/31178118] was used to import the output of Cellranger count using the *Read10X* function for further data analysis. The low-quality cells with few expressed genes (n<200) were removed. After filtering, a total of 6,326 cells expressing at least 200 genes with mitochondrial counts of less than 10% were selected for the analysis (**Supplementary Figures 5C** and **5D**). Data was then batch corrected between 3 samples using 30 dimensions as input parameter. Clustering was performed using resolution 0.06 and cells were visualized using tSNE plots with dimension of 20 and perplexity of 30. SingleR R package was used for determining classes of cells with MouseRNASeq as the reference dataset (66, 67). For further refinement of clusters identified as T-cells, gene markers that are associated with different CD4+ and CD8+ T cells were plotted and the cells that expressed these markers were classified as such. Similarly, cells identified as macrophages were further resolved in subtypes based on respective gene marker expression. Differential expression was performed using Seurat’s default non-parametric Wilcoxon rank sum test and statistically significant genes (p < 0.05) that were different between the samples in assigned cell type clusters were used for pathway analyses using Reactome (40). The data was submitted to NCBI GEO database under accession number GSE159553.

### Statistics

Mice in the experiments were randomly assorted into cages by Animal Facility staff and not further randomized. Data acquisition and analysis were not blinded. All statistical analyses were performed with PRISM V8.2.1 and R V3.5.1. Each individual data point was sampled independently. Two-tailed Mann Whitney Rank tests were used to compare differences between groups. Log Rank test was used to compare survival between two groups in challenge survival studies. Bonferroni corrections were used to adjust for multiple comparisons.

## Data availability statement

The data presented in the study are deposited in the NCBI GEO database, accession number GSE159553. Further inquiries can be directed to the corresponding authors.

## Ethics statement

The animal study was reviewed and approved by Institutional Animal Care and Use Committee of the Wistar Institute.

## Author contributions

NT, ZX, DK and DW conceptualized the project. NT, ZX, DK and DW planned the experiments. NT, ZX, MH, SW, KL, NC, TK, YW, ET-R, DP, XZ, MW, SM, and AK conducted the experiments. TS contributed crucial reagents or equipment. NT, ZX, DK, and DW analyzed the data. NT, ZX, SM, AK, DK and DW wrote the paper. NT and ZX share co-first authorship due to their equal contributions to the project, with the order based on length of involvement. All authors contributed to the article and approved the submitted version
