# Supplementary material for: Engineered antibody cytokine chimera synergizes with DNA-launched nanoparticle vaccines to potentiate melanoma suppression in vivo
Source: Front Immunol. 2023 Feb 23;14:1072810. doi: 10.3389/fimmu.2023.1072810 (PMC9997082; doi:10.3389/fimmu.2023.1072810)
Supplement: Supplementary file 1 [file DataSheet_1.docx]

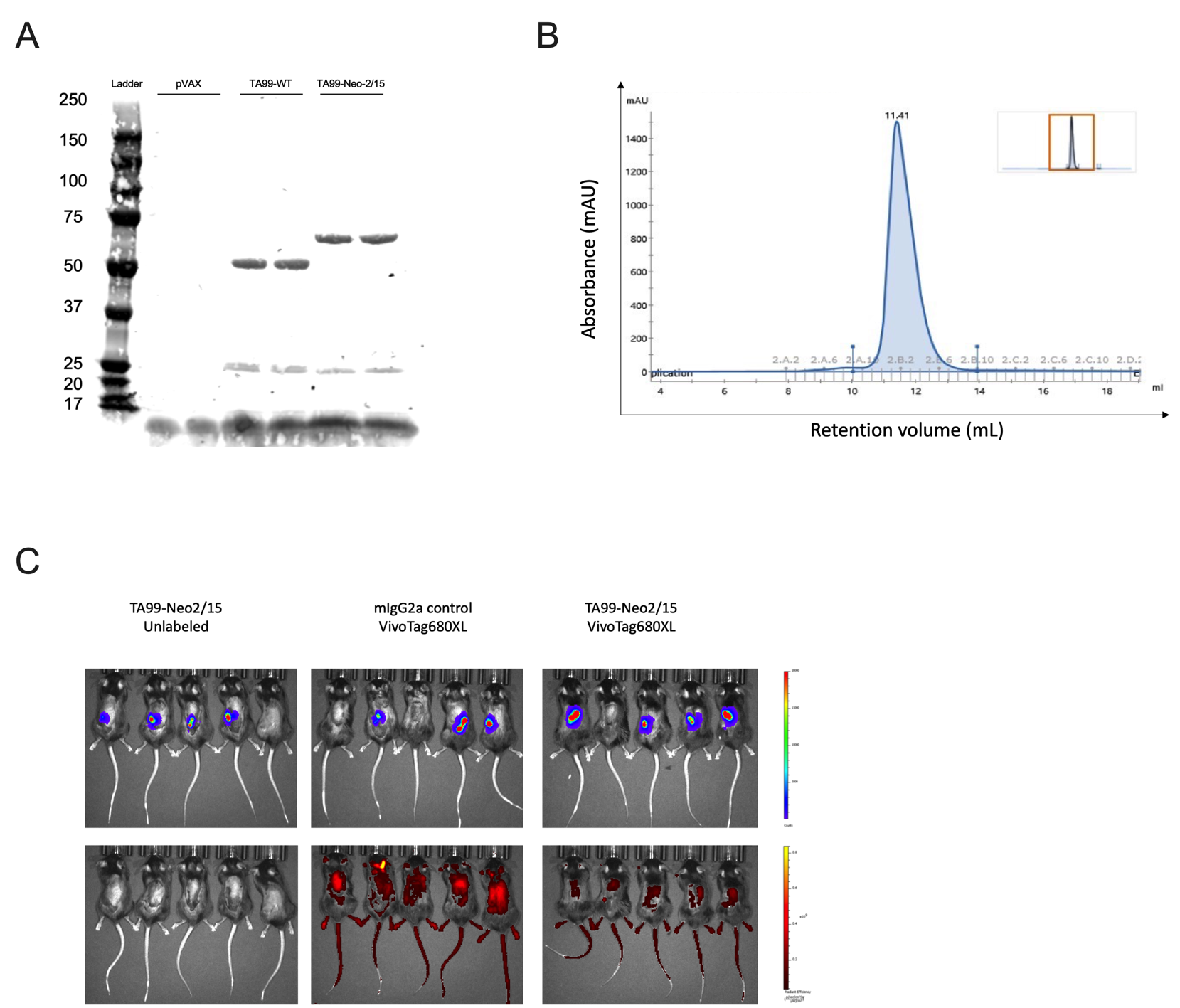


**Supplementary Figure 1.** **In vitro and in vivo characterization of ACC TA99-Neo2/15 (Related to Fig. 1).** **A.** Reducing SDS-PAGE analysis comparing the migration patterns of TA99-WT to TA99-Neo-2/15 compared to the backbone vector pVAX transfection supernatant. **B.** SECMAL trace of recombinant TA99-Neo2/15 following protein G column purification**.** **C**. IVIS to determine *in vivo* colocalization of tumor with VivoTag680XL-conjugated ACC TA99-Neo2/15 or murine IgG2a isotype control. C57BL/6 mice were inoculated with 5x10^5^ B16F10.Luc cells subcutaneously followed by administration of unlabeled TA99-Neo2/15, VivoTag680XL-conjugated TA99-Neo2/15, or VivoTag680XL-conjugated IgG2a control (2nmol) on D8. The mice were imaged on D9 to visualize location of tumor *in vivo* with luminescence (top panel) or distribution of antibodies with fluorescence (bottom panel).

**
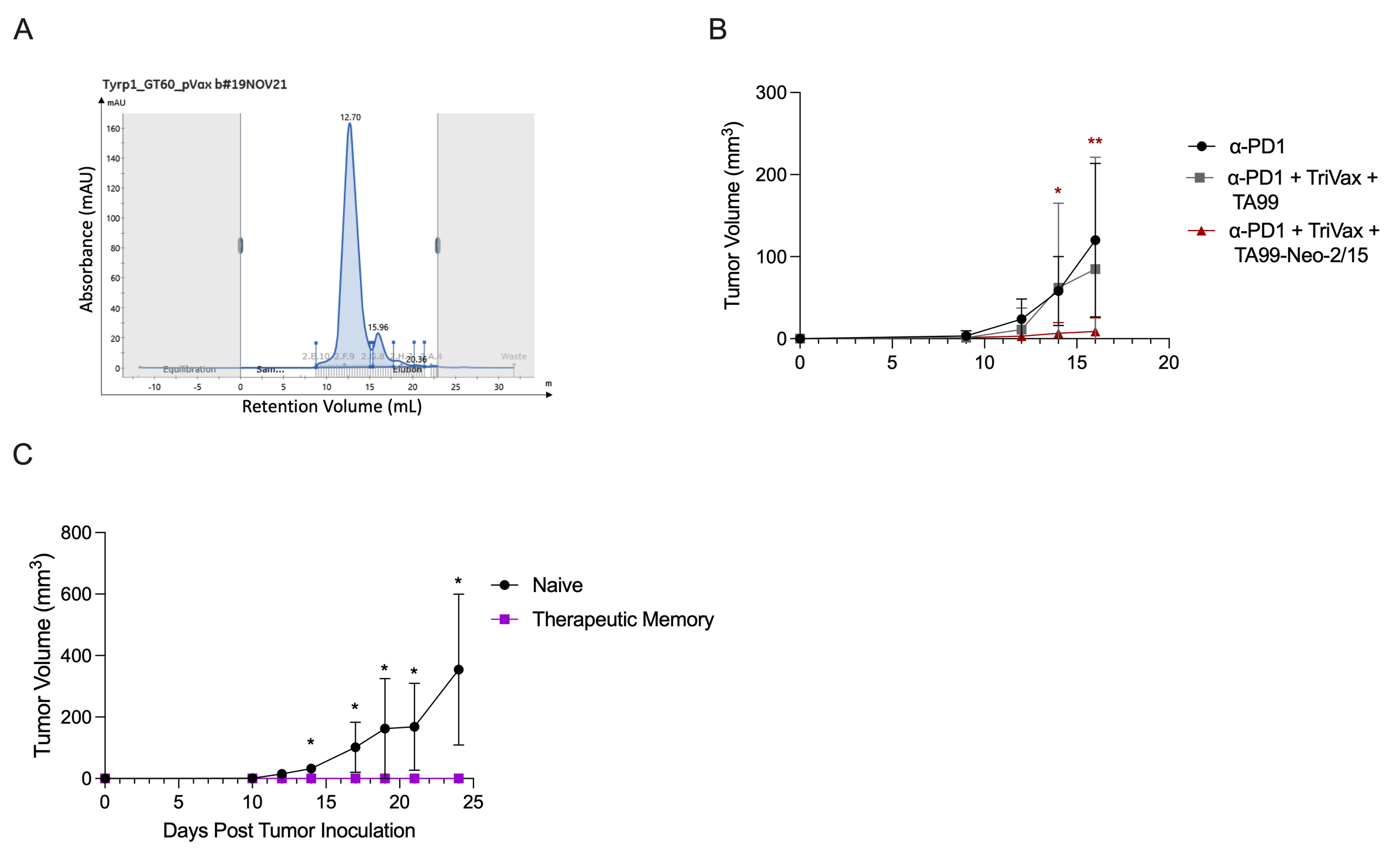
**

**Supplementary Figure 2.** ***In vivo* characterization of TA99-based immunotherapies and DLnano-vaccines (Related to Fig. 1)**. **A.** SEC trace showing the assembly of DLnano_LS_Tyrp1_455_ following lectin-column purification. **B.** Tumor volume following the initial tumor challenge of mice treated with anti-PD1 alone (200ug), or anti-PD1 (200ug) + WT TA99 (50ug) + TriVax, or anti-PD1 (200ug) + TA99-Neo2/15 (50ug) + TriVax. **C.** Tumor volume following rechallenge in mice that survived the initial challenge in **B** or in 5 naïve mice. N=10 for **B.** Each dot represents an animal; error bars represent standard deviation. Non-parametric Kruskal-Wallis test adjusted for multiple comparisons was used in **B**; color of asterisk represents group that is significant relative to control treatment anti-PD1; two-tailed Mann-Whitney rank test was used to compare differences between groups in **C**; log-rank tests were used to compare between differences in all survival curves; *p<0.05, **p<0.01.

**
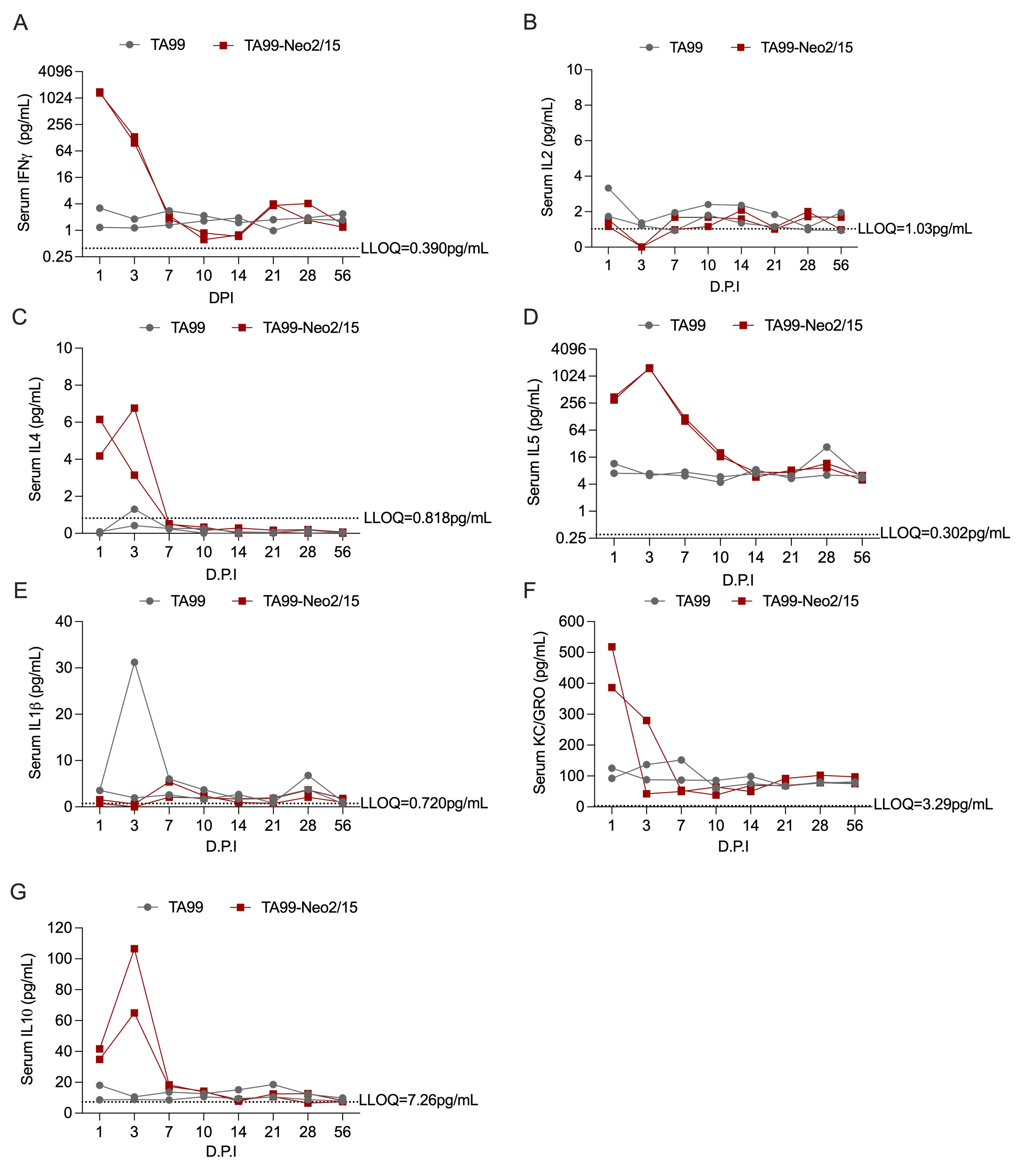
**

**Supplementary Figure 3.** **Systemic proinflammatory responses in mice treated with ACC TA99-Neo2/15** **(Related to Fig. 1)**. **A**-**G**. Time-course of serum cytokine levels post 100µg administration of antibody construct intraperitoneally to C57BL/6 mice (n=5/group); 2 sera pools were analyzed for each treatment condition. Serum levels of IFNγ (**A**), IL-2 (**B**), IL-4 (**C**), IL-5 (**D**), IL-1β (**E**), KC/GRO (**F**), and IL-10 (**G**) were monitored up to 56 days post administration.


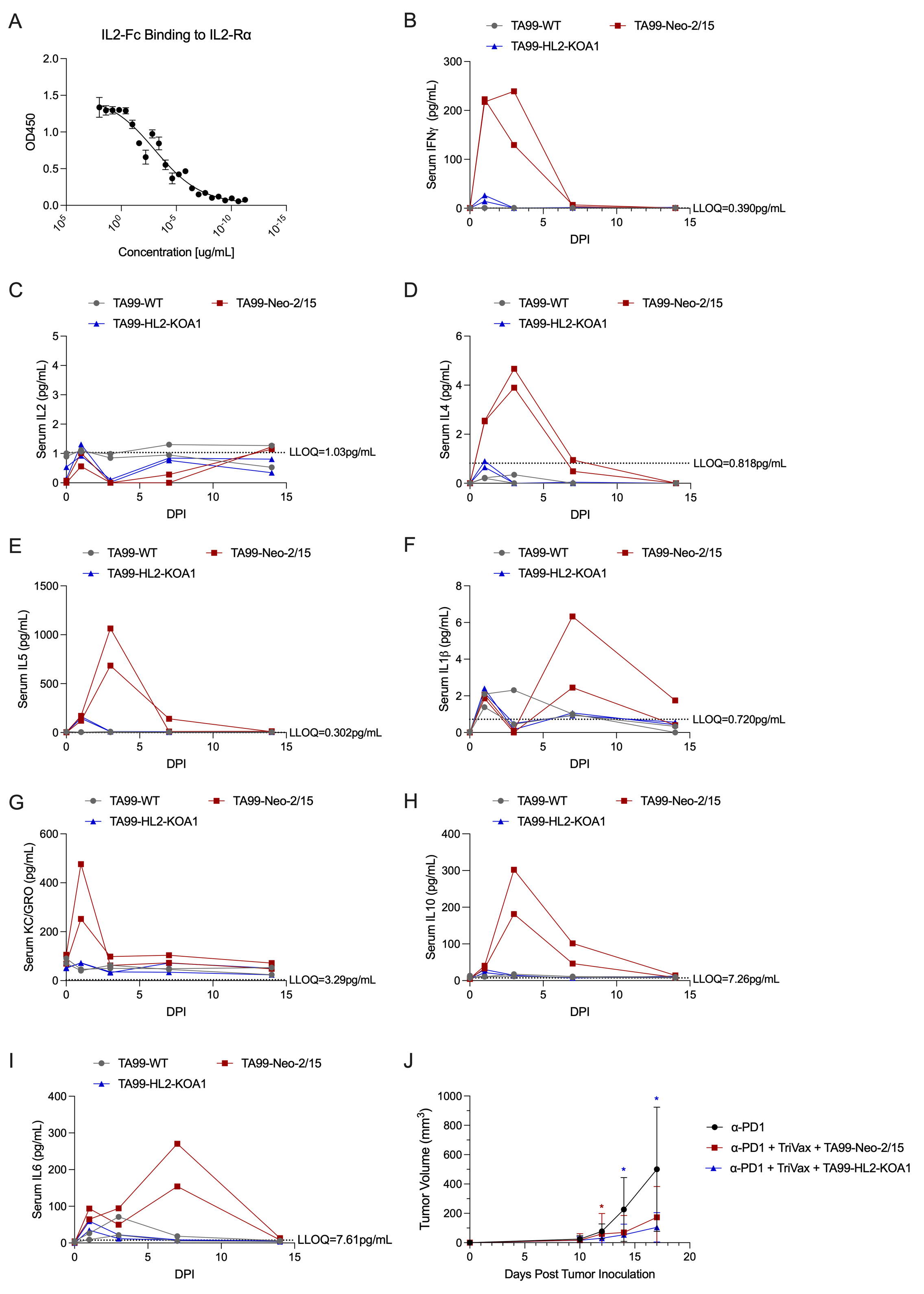


**Supplementary Figure 4.** **Pharmacokinetic and pharmacodynamic characterizations of novel ACC TA99-HL2-KOA1 (Related to Fig. 2). A.** Binding of human IL2-Fc to recombinant human IL2-Rα as measured by binding ELISA. **B**-**I**. Time course of serum cytokine levels post 100µg administration of ACCs intraperitoneally to C57BL/6 mice. Two sera pools were used for the analysis. Serum levels of IFNγ (**B**), IL-2 (**C**), IL-4 (**D**), IL-5 (**E**), IL-1β (**F**), KC/GRO (**G**), IL-10 (**H**), and IL-6 (**I**) were monitored up to 14 days post administration. **J.** Tumor volume following tumor challenge of mice treated with anti-PD1 alone (200ug), or anti-PD1 (200ug) + TA99-Neo2/15 (50ug) + TriVax, or anti-PD1 (200ug) + TA99-HL2-KOA1 (50ug) + TriVax. N=10 for panel **J**; error bars represent standard deviation. Non-parametric Kruskal-Wallis test adjusted for multiple comparisons was used in **D**; color of asterisk represents group that is significant relative to control treatment anti-PD1. *p<0.05.

**Supplementary Figure 5.** **Analysis of splenic CD4+ T cell responses following administration of DLnano-vaccine and immunotherapy in tumor bearing mice and analysis of tumor-infiltrating lymphocytes with transcriptomics (Related to Fig. 3). A** and **B**. DLnano-vaccine induced LS-specific splenic IFNγ+ CD4+ T-cell responses as determined by ICS (**A**) and ELIspot (**B**). **C** and **D.** Filtering criteria (**C**) and corresponding number of cells filtered (**D**) in determination of cells that would be used for downstream clustering and gene expression profile analysis. N=5 mice/group for **A** and **B**; N=5 mice/ group were pooled for transcriptomic analyses in **C**-**D**. each dot represents an individual animal. Error bar represents standard deviation. Two-tailed Mann-Whitney Rank test used to compare groups; p-values were adjusted for multiple comparison for **A** and **B**. *p<0.05.

**Supplementary Figure 6.** **Transcriptomic analyses indicate increased effector functions in CD4+, CD8+ T cells and NK cells following Trp2Vax and TA99-based immunotherapy treatments**. **A**-**D**. Expression of CD4 (**A**), CD8a (**B**), CD8b1 (**C**) and Foxp3 (**D**) in sample integrated TSNE plot shown in **Fig. 3E**. **E**. Expression of TOX, XCL1, IL-7R, CD28, PRF1 and GZMB in sample integrated TSNE plot shown in **Fig. 3E**. **F**. Identification of naïve, stem-like, and terminally differentiated CD8+ T cells according to the markers shown in **E**. **G.** Relative frequencies of naïve, stem-like, and terminally-differentiated CD8+ T cells in mice treated with only anti-PD1, with anti-PD1+ Trp2Vax + WT TA99 or with anti-PD1+ Trp2Vax + TA99-HL2-KOA1. **H-J.** Top upregulated and downregulated genes in intratumoral CD4+ T cells (**H**), CD8+ T cells (**I**) and NK cells (**J**) in mice treated with either anti-PD1+ Trp2Vax + TA99 or anti-PD1+ Trp2Vax + TA99-HL2-KOA1 versus corresponding cell types from mice treated with only anti-PD1. N=5 mice/group were pooled for transcriptomic analyses.

**Supplementary Figure 7.** **Transcriptomic analyses indicate increased effector functions in B cells, dendritic cells, macrophages, and monocytes following Trp2Vax and TA99-based immunotherapy treatments A**-**D.** Top upregulated and downregulated genes in intratumoral B cells (**A**), DCs (**B**), macrophages (**C**) and monocytes (**D**) in mice treated with either anti-PD1+ Trp2Vax + TA99 or anti-PD1+ Trp2Vax + TA99-HL2-KOA1 versus corresponding cell types from mice treated with only anti-PD1. N=5 mice/ group were pooled for transcriptomic analyses in this Figure.

**Supplementary Figure 8.** **Treatment with Trp2Vax and Immunotherapy resulted in downregulation of heat shock responses in tumor infiltrating immune cells. (Related to Fig. 4). A.** Mapping of the HSF-1 heat shock responsive pathway. **B** and **C**. Assessment of fold changes of different genes in the HSF-1 heat shock responsive pathway amongst listed TIL cell types from mice treated with anti-PD1+ Trp2Vax + TA99 (**B**) or anti-PD1+ Trp2Vax + TA99-HL2-KOA1 (**C**) relative to corresponding cell types in mice treated with only anti-PD1. N=5 mice/ group were pooled for transcriptomic analyses in this Figure.

**Supplementary Figure 9.** **Analysis of tumor infiltrating CD4+ Foxp3+ Tregs with transcriptomics (Related to Fig. S6).** **A**. Map of EML and NUDC pathway in mitotic spindle formation by Reactome. **B**. Top downregulated genes in intratumoral Tregs in mice treated with anti-PD1+ Trp2Vax + TA99-HL2-KOA1 versus intratumoral Tregs from mice treated with only anti-PD1 or with anti-PD1+ Trp2Vax + WT TA99. N=5 mice/ group were pooled for transcriptomic analyses in this Figure.
